# Fetal hemoglobin in umbilical cord blood in preeclamptic and normotensive pregnancies: A cross-sectional comparative study

**DOI:** 10.1371/journal.pone.0176697

**Published:** 2017-04-28

**Authors:** Zahra Masoumi, Mary Familari, Karin Källén, Jonas Ranstam, Per Olofsson, Stefan R. Hansson

**Affiliations:** 1 Department of Clinical Sciences Lund, Division of Obstetrics and Gynecology, Lund University, Lund, Sweden; 2 School of Biosciences, University of Melbourne, Parkville, Australia; 3 Center for Reproductive Epidemiology, Department of Clinical Sciences Lund, Lund University, Lund, Sweden; 4 Department of Clinical Sciences Lund, Division of Orthopedics, Faculty of Medicine, Lund University, Lund, Sweden; 5 Department of Clinical Sciences Malmö, Division of Obstetrics and Gynecology, Lund University, Skåne University Hospital, Malmö, Sweden; Hungarian Academy of Sciences, HUNGARY

## Abstract

Preeclampsia (PE) is associated with increased fetal hemoglobin (HbF) in the maternal circulation but its source is unknown. To investigate whether excessive HbF is produced in the placenta or the fetus, the concentration of HbF (*c*HbF) in the arterial and venous umbilical cord blood (UCB) was compared in 15825 normotensive and 444 PE pregnancies. The effect of fetal gender on *c*HbF was also evaluated in both groups. Arterial and venous UCB sampled immediately after birth at 36–42 weeks of gestation were analyzed for total Hb concentration (*c*tHb) (g/L) and HbF% using a Radiometer blood gas analyzer. Non-parametric tests were used for statistical comparison and *P* values < 0.05 were considered significant. Our results indicated higher *c*HbF in venous compared to arterial UCB in both normotensive (118.90 vs 117.30) and PE (126.75 vs 120.12) groups. In PE compared to normotensive pregnancies, a significant increase was observed in arterial and venous *c*tHb (171.00 vs 166.00 and 168.00 vs 163.00, respectively) while *c*HbF was only significantly increased in venous UCB (126.75 vs 118.90). The pattern was similar in both genders. These results indicate a substantial placental contribution to HbF levels in UCB, which increases in PE and is independent of fetal gender, suggesting the elevated *c*HbF evident in PE results from placental dysfunction.

## Introduction

Preeclampsia (PE) is a leading cause of fetal and maternal mortality and morbidity, affecting 3–8% of pregnancies worldwide [1–3]. PE evolves in two stages; the first stage is initiated by inadequate placentation and insufficient remodeling of the uterine spiral arteries [4]. In stage two, the clinical manifestations occur after 20 weeks of gestation. Several factors, including free fetal hemoglobin (HbF), leak from the placenta into the maternal circulation, inducing inflammation and oxidative stress (OS) causing widespread vascular endothelial damage, a hallmark of PE [5–8].

Preeclamptic placentas have increased HbF gene expression and accumulation of free HbF protein [9]. Free HbF has been shown to cause OS and damage the placenta barrier [10, 11] due to its high redox potential [5, 12]. As a consequence, leakage of HbF into the maternal circulation has been reported as early as the first trimester in pregnant women who later develop PE [11]. Furthermore, in term pregnancies, the level of HbF in the maternal plasma correlates with the severity of the disease [10]. Although its mechanism of contribution to the pathophysiology of PE has been studied and reviewed [13, 14], the source of the free HbF has not been investigated.

The increased HbF in the maternal plasma has been demonstrated among PE women independent of fetal gender. Several studies have reported Hb levels among newborns to be dependent on fetal gestational age and gender [15, 16] while increased Hb has been associated with fetal mortality [17]. Other studies have suggested gender-specificity in maternal adaptation to pregnancy [18], placental function and gene expression [19, 20] and occurrence of PE [21, 22].

The placenta plays various roles during fetal development; from mediating gas and nutrition exchange to producing several hormones and growth factors [23]. It is also a hematopoietic organ, bearing hematopoietic stem/progenitor cells (HSPCs) [24] and contributing to extramedullary fetal hematopoiesis and erythropoiesis [24–29]. Parameters measured in the venous UCB of the fetoplacental circulation can be ascribed to placental function [30].

The aim of the study was to evaluate if the increased synthesis and accumulation of free HbF previously observed in PE placentas is related to fetal gender and altered placental function. The placental contribution of HbF was estimated by comparing its concentration in venous and arterial umbilical cord blood (UCB) in PE vs normotensive pregnancies.

## Methods

### Blood analysis

Arterial and venous UCB gas analysis at birth has been a routine procedure at the Skåne University Hospital maternity units in Malmö and Lund for the last three decades. During the period 2001–2010, blood samples were obtained in 2-mL pre-heparinized syringes immediately after birth and analyzed within 15 minutes using Radiometer ABL 735 blood gas analyzers (Radiometer A/S, Copenhagen, Denmark). In addition to blood gases, the analyzer measures the pH by potentiometry as well as various compounds such as the total hemoglobin concentration (*c*tHb) (g/L) and HbF% by spectrophotometry at 37°C. In order to obtain optical clarity, the blood gas analyzer hemolyzes the blood samples by ultrasonication, which prevents distinguishing free Hb in the plasma from the intracellular Hb released due to ultrasonication. As HbF and adult hemoglobin have different molecule structures and light absorbance spectra, they can be measured at different wavelengths. The *c*tHb includes fractions of oxy-, carboxy-, deoxy- and methemoglobin and the HbF concentration (*c*HbF) (g/L) can be derived from *c*HbF = HbF% × *c*tHb. All the obtained data was transferred to an electronic database including a report of analysis quality for each parameter and personal identification number for each analysis. The origin of sampling, i.e. “artery” or “vein”, was also indicated for each sample at analysis. The placental contribution to the UCB Hb concentration was calculated by deducting the arterial value from that of the venous in paired samples, where both arterial and venous Hb values were available. These factors were indicated as VA*c*tHb and VA*c*HbF for tHb and HbF, respectively.

### Study group

After the study was approved by the Regional Ethics Committee in Lund, Sweden (Dnr 2009/222), the laboratory data was paired with the clinical data from the regional Perinatal Revision South Register for the aforementioned period. Any analysis with quality check error was excluded and each blood analysis was validated for the vessel of the origin by the criterion that the venous pH should be at least 0.02 units higher than in the artery [31]. Out of a total number of 44,423 patients merged from Lund and Malmö databases, the final validated sample population consisted of 16,269 patients with ensured identifications, complete panels of maternal and fetal clinical data, and paired arterial and venous UCB pH determinations with *c*tHb and HbF% in the artery and/or vein.

PE was defined as BP ≥ 140/90 mmHg plus proteinuria ≥ 300 mg/L and severe PE as BP ≥ 160/110 mmHg [32, 33].

### Statistical analyses

The Mann-Whitney *U* test and the Wilcoxon signed-rank test for matched pairs were used to compare the distribution of continuous variables among all and paired samples, respectively. Spearman’s rank correlation (rho) were used to calculate the correlation between variables and the 95% confidence interval (CI) for the correlation value was determined by bootstrapping for 1000 samples. Finally, The Chi-Square test was employed for comparisons of categorical variables. Values are reported as median ± 95% CI. The statistics was performed using SPSS computer software (SPSS Statistics for Macintosh, Version 23.0. Armonk, IBM Corp., NY) and *p* values < 0.05 were considered statistically significant.

## Results

The distribution of the blood samples based on maternal condition and fetal gender is presented in Table 1. Among the 16,269 UCB samples with *c*HbF data, 444 (2.72%) were diagnosed with PE; 393 (2.41%) with mild-moderate PE cases and 51 (0.31%) with severe PE patients. As the number of severe PE samples was considered too small, the two PE subgroups were merged and compared with normotensive pregnancies. The two groups were also examined for gestational age differences on HbF production. No major differences were observed in gestational age in the two groups (Median: 39; interquartile range: 38–40 weeks, in both groups).

**Table 1:** Distribution of maternal condition and fetal gender in the study population.

Maternal condition	N	*c*tHb (N)	*c*HbF (N)	Gender N (%)	
				Male	Female
**Normotensive**	15,825	Artery only (8)	Artery only (414)	8231 (97.16%)	7594 (97.38%)
		Vein only (115)	Vein only (12,280)		
		Paired ^a^ (15,698)	Paired ^a^ (3127)		
		Missing (4)	Missing (4)		
**Mild-moderate PE**	393	Artery only (0)	Artery only (19)	209 (2.47%)	184 (2.36%)
		Vein only (1)	Vein only (302)		
		Paired ^a^ (392)	Paired ^a^ (72)		
**Severe PE**	51	Artery only (0)	Artery only (1)	31 (0.37%)	20 (0.26%)
		Vein only (2)	Vein only (44)		
		Paired ^a^ (49)	Paired ^a^ (6)		
**Total**	16,269	Artery only (8)	Artery only (434)	8471 (52.07%)	7798 (47.93%)
		Vein only (118)	Vein only (12,626)		
		Paired ^a^ (16,139)	Paired ^a^ (3205)		
		Missing (4)	Missing (4)		

Number of samples with concentration of total hemoglobin (*c*tHb) and fetal Hb (*c*HbF) from arterial and venous UCB have been indicated in relevance to fetal gender and maternal condition (normotensive, mild-moderate preeclampsia (PE) and severe PE).

a. Both arterial and venous values were available.

### Comparing arterial and venous UCB *c*tHb and *c*HbF

Statistically significant correlation was found between the arterial and venous values of *c*tHb (rho: 0.86, 95% CI: 0.85–0.88) and *c*HbF (0.83, 0.81–0.84) using Spearman’s rank correlation (*P* = 0.01). In both normotensive and PE pregnancies, the median arterial *c*tHb was higher than the venous (relative increase: 1.8%, 95% CI; 1.8–2.4%) whereas the median *c*HbF was higher in the vein rather than the artery (2.0%, 2.0–2.1%); both differences were statistically significant (*P* < 0.001, exact median ± 95% CI values for each factor are indicated in Table 2).

**Table 2:** Median values with 95% confidence intervals (CI) for arterial, venous and veno-arterial difference of (VA) *c*tHb and *c*HbF.

	Maternal condition	Arterial *c*tHb (g/L)	95% CI		Venous *c*tHb (g/L)	95% CI		Arterial *c*HbF (g/L)	95% CI		Venous *c*HbF (g/L)	95% CI		VA*c*tHb (g/L)	95% CI		VA*c*HbF (g/L)	95% CI	
			Lower Bound	Upper Bound		Lower Bound	Upper Bound		Lower Bound	Upper Bound		Lower Bound	Upper Bound		Lower Bound	Upper Bound		Lower Bound	Upper Bound
**Paired**^**a**^	Normot-ensive	166.00	166.00	166.00	163.00	162.00	163.00	117.45	116.80	118.14	119.88	119.28	120.45	-3.00	-4.00	-3.00	2.80	2.40	3.24
	PE	171.00	169.00	173.00	168.00	166.00	170.00	122.59	117.92	127.97	128.35	123.54	130.24	-3.00	-5.00	-1.00	3.56	1.31	6.32
**All**	Normot-ensive	166.00	166.000	166.0	163.00	162.00	163.00	117.30	116.64	117.86	118.90	118.50	119.28	NA^b^	NA	NA	NA	NA	NA
	PE	171.00	169.00	173.00	168.00	167.00	170.00	120.12	117.81	124.80	126.75	124.82	129.20	NA	NA	NA	NA	NA	NA

The values for arterial and venous *c*tHb and *c*HbF (± 95% CI) as well as the veno-arterial difference of (VA) *c*tHb and *c*HbF (± 95% CI) are demonstrated in relevance to the maternal condition (normotensive and PE) for samples with both arterial and venous values available for each patient (paired) as well as all the samples (all).

a. Both arterial and venous values were available.

b. NA: Not available. It was not possible to calculate these values among all the samples, as the arterial or venous value for *c*tHb and/or *c*HbF was missing in some of the samples (refer to Table 1).

### Comparing normotensive vs PE values of the arterial and venous *c*tHb and *c*HbF

**Fig 1:**
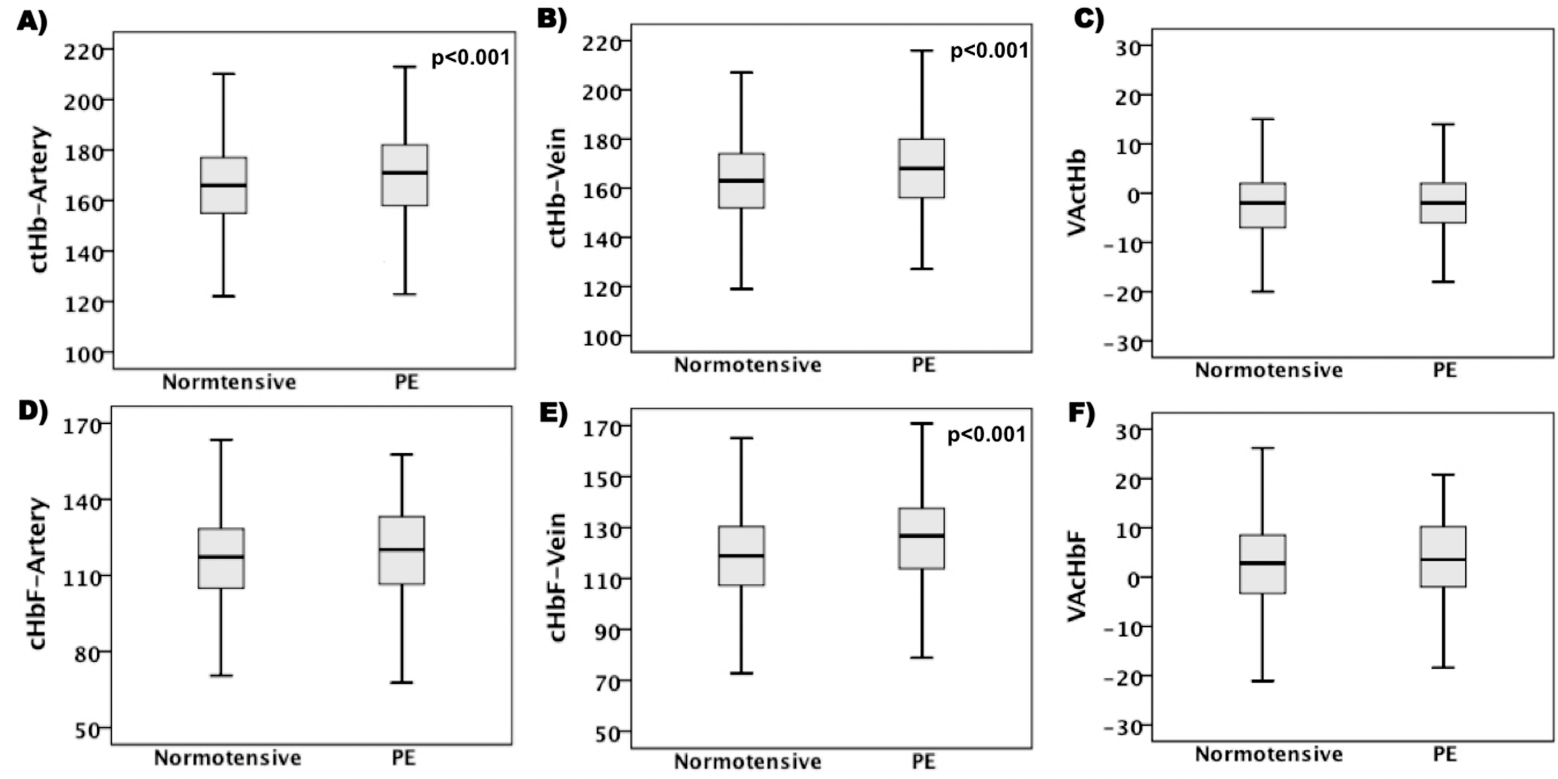
Comparison of hemoglobin (Hb) values from umbilical cord blood of normotensive and preeclamptic (PE) pregnancies. (A) Arterial concentration of total Hb (ctHb), (B) venous ctHb, (C) veno-arterial difference of (VA) ctHb, (D) arterial concentration of fetal Hb (cHbF), (E) venous cHbF and (F) VAcHbF. Boxplots showing median, first and third quartiles and maximum and minimum values and levels of significance.

To investigate the influence of PE on the arterial and venous *c*tHb and *c*HbF, the distribution of all the values from normotensive and PE groups were compared using Mann-Whitney *U* test (Table 2). Compared to normotensive pregnancies, the UCB from the PE samples had statistically significantly higher arterial and venous *c*tHb (relative increase: 3.0%, 95% CI: 1.0–4.2% and 3.0%, 2.4–4.2%, respectively) (Fig 1A and 1B) and venous *c*HbF (6.0%, 5.0–8.0%) (Fig 1E). There were no statistically significant differences in median arterial *c*HbF (Fig 1D), albeit estimated with some uncertainty as indicated by the 95% CI (Table 2). The median and 95% CI values of VA*c*tHB or VA*c*HbF were not significantly different between PE and normotensive pregnancies either (Fig 1C and 1F).

### Evaluating gender-specific effect of PE on *c*tHb and *c*HbF

**Fig 2:**
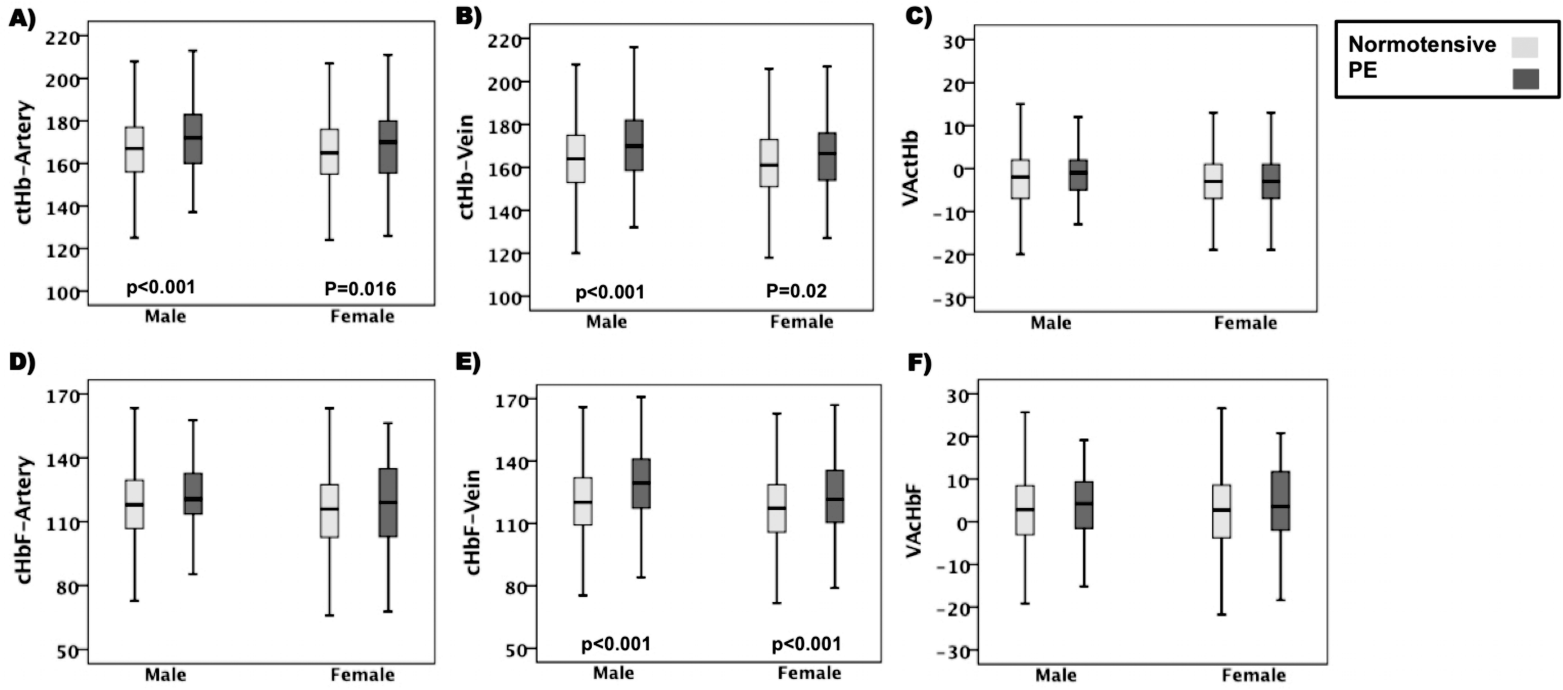
Intra-gender comparison of UCB Hb values in normotensive vs PE pregnancies. (A) arterial UCB ctHb, (B) venous UCB ctHb, (C) VA ctHb, (D) arterial UCB cHbF, (E) venous UCB cHbF and (F) VAcHbF. Boxplots showing median, first and third quartiles and maximum and minimum values and levels of significance.

The effect of fetal gender on Hb values was studied separately in normotensive and PE groups using Mann-Witney *U* test. The arterial and venous UCB *c*tHb and *c*HbF were statistically significantly (*P < 0*.*001*) higher (1.2%, 1.8%, 1.7% and 2.3%, respectively) in male versus female infants from normotensive pregnancies and a similar pattern was observed among PE pregnancies. To elucidate gender-specific changes, intra-gender analysis was performed between normotensive and PE pregnancies. Arterial and venous *c*tHb and venous *c*HbF medians were statistically significantly (*P < 0*.*02*) higher in both male (2.9%, 3.6% and 7.8%, respectively) and female newborns (3.0%, 3.1% and 3.6%, respectively) affected by PE (Fig 2A, 2B and 2E). However, the differences between normotensive vs PE arterial *c*HbF, VA*c*tHB and VA*c*HbF medians (Fig 2D, 2C and 2F) were not statistically significant among male or female newborns.

## Discussion

To our knowledge, this is the first study to recognize placental contributions to the UCB and distinguish it from the fetal endowment by comparing *c*tHb and *c*HbF in venous and arterial UCB from normotensive and PE pregnancies. The prevalence of PE in our database, which was collected over 10 years, was in agreement with previous data from Sweden. The number of severe PE cases was typically low for Sweden preventing us from including subgroups of PE.

The clinical relevance of our findings lies in the fact that arterial UCB provides an indication of cellular processes in the fetus, hence an indication of fetal health status. Our results showing significantly higher arterial *c*tHb may reflect active fetal erythropoiesis. This is in line with the present findings showing increased arterial UCB *c*tHb in PE and other studies reporting a higher proportion of nucleated reticulocytes [29, 34, 35] and younger population of red blood cells [36] in the fetuses from PE pregnancies that counteract the chronic hypoxic conditions, which induces erythropoietin synthesis and erythropoiesis [37, 38].

Our analyses showed significantly higher venous UCB *c*HbF compared to the arterial in both normotensive and PE cases. Interestingly, the venous UCB *c*HbF was significantly higher (5–8%) in PE compared to normotensive pregnancies. Even though a general biological variation is about 10%, the increased *c*HbF may play a role in the pathophysiology of PE. As the damage observed in the placental barrier in PE [10, 11], may alter the fluid homeostasis, the increase in venous *c*HbF may inaccurately be attributed to fluid loss across the placenta and decreased venous plasma volume after placental passage. However, this is unlikely because the *c*tHb was lower in the venous UCB compared to arterial in both normotensive and PE pregnancies. As the venous, but not arterial, UCB represents contributions from the placenta, our results suggest a possible role for the placenta in altering the HbF levels. Interestingly, previous studies have described changes in the localization of placental hematopoietic stem/ progenitor cells (HSPCs) [39] and increased placental HbF mRNA and protein [9] in PE suggesting altered placental erythropoiesis as a plausible cause of the increased venous *c*HbF.

Gender-based differences (approximately 10%) in Hb values are clinically significant among adults [40], and the positive effect of testosterone in males [41–43] seems to be as important as the negative influence of menstruation in females [44] in intensifying this difference. Other studies have reported gender is an important factor affecting Hb concentration already during infancy [16, 45]. We observed significantly higher arterial and venous *c*tHb in males than females that led us to perform intra-gender analyses between PE and normotensive groups. Both males and females demonstrated an increase in arterial and venous *c*tHb as well as venous *c*HbF, showing that there was no gender-specific pattern for Hb alteration in PE. Interestingly, a gender-specific pattern of increased risk of PE, abruptio placenta and pre-term birth has been reported in pregnancies with a male fetus [21, 46–49]. However, it has also been reported that a female fetus increases the risk of pre-term delivery (<37 gestational weeks) in PE pregnancies [22]. Inter-gender differences in Hb concentration may play a role in upregulating protective scavenger proteins such as hemopexin, haptoglobin and the rate limiting degradation enzyme heme-oxygenase 1, and thereby provide a protection against PE. Higher Hb in male fetuses could also be a risk factor per se by contributing to the elevated free Hb levels observed in PE. Further investigations are required to elucidate how these gender-specific differences in erythropoiesis may affect PE manifestations.

In conclusion, our findings confirm previous reports regarding increased Hb and erythrocytes in fetuses from PE pregnancies. Importantly, our results also show a significant increase in venous *c*HbF in the UCB, particularly in PE pregnancies. Accordingly, we propose a placental regulation of HbF that may be compromised in PE independent of fetal gender. Elucidating the exact underlying mechanisms requires further investigation on sample populations with higher number of severe PE cases where free HbF can be distinguished from its intracellular form.

## Supporting information

S1 Table The raw data used in the study. All the raw data used in the study including the arterial and venous *c*tHb, HbF%, *c*HbF, maternal condition and its severity as well as fetal gestational age and gender is included in this table.
(XLSX)

## References

[1] Hutcheon JA, Lisonkova S, Joseph KS. Epidemiology of pre-eclampsia and the other hypertensive disorders of pregnancy. Best Pract Res Clin Obstet Gynaecol. 2011;25(4):391–403. doi: 10.1016/j.bpobgyn.2011.01.006 21333604 10.1016/j.bpobgyn.2011.01.006

[2] Duley L. The global impact of pre-eclampsia and eclampsia. Semin Perinatol. 2009;33(3):130–7. doi: 10.1053/j.semperi.2009.02.010 19464502 10.1053/j.semperi.2009.02.010

[3] WHO. WHO recommendations for Prevention and treatment of pre-eclampsia and eclampsia. Geneva, Switzerland: World Health Organization; 2011. 23741776

[4] Roberts JM, Cooper DW. Pathogenesis and genetics of pre-eclampsia. Lancet. 2001;357(9249):53–6. 11197372 10.1016/s0140-6736(00)03577-7

[5] Hansson SR, Gram M, Akerstrom B. Fetal hemoglobin in preeclampsia: a new causative factor, a tool for prediction/diagnosis and a potential target for therapy. Curr Opin Obstet Gynecol. 2013;25(6):448–55. doi: 10.1097/GCO.0000000000000022 24185004 10.1097/GCO.0000000000000022

[6] Ouyang YQ, Li SJ, Zhang Q, Cai HB, Chen HP. Interactions between inflammatory and oxidative stress in preeclampsia. Hypertens Pregnancy. 2009;28(1):56–62. doi: 10.1080/10641950802233064 19165670 10.1080/10641950802233064

[7] Fialova L, Kalousova M, Soukupova J, Malbohan I, Madar J, Frisova V, et al Markers of inflammation in preeclampsia. Prague Med Rep. 2004;105(3):301–10. 15782556

[8] Sanchez-Aranguren LC, Prada CE, Riano-Medina CE, Lopez M. Endothelial dysfunction and preeclampsia: role of oxidative stress. Front Physiol. 2014;5:372 PubMed Central PMCID: PMCPMC4193194. doi: 10.3389/fphys.2014.00372 25346691 10.3389/fphys.2014.00372 PMC4193194

[9] Centlow M, Carninci P, Nemeth K, Mezey E, Brownstein M, Hansson SR. Placental expression profiling in preeclampsia: local overproduction of hemoglobin may drive pathological changes. Fertil Steril. 2008;90(5):1834–43. PubMed Central PMCID: PMCPMC2628488. doi: 10.1016/j.fertnstert.2007.09.030 18166190 10.1016/j.fertnstert.2007.09.030 PMC2628488

[10] May K, Rosenlof L, Olsson MG, Centlow M, Morgelin M, Larsson I, et al Perfusion of human placenta with hemoglobin introduces preeclampsia-like injuries that are prevented by alpha1-microglobulin. Placenta. 2011;32(4):323–32. doi: 10.1016/j.placenta.2011.01.017 21356557 10.1016/j.placenta.2011.01.017

[11] Anderson UD, Olsson MG, Rutardottir S, Centlow M, Kristensen KH, Isberg PE, et al Fetal hemoglobin and alpha1-microglobulin as first- and early second-trimester predictive biomarkers for preeclampsia. Am J Obstet Gynecol. 2011;204(6):520 e1–5. 21439542 10.1016/j.ajog.2011.01.058

[12] Reeder BJ. The redox activity of hemoglobins: from physiologic functions to pathologic mechanisms. Antioxid Redox Signal. 2010;13(7):1087–123. doi: 10.1089/ars.2009.2974 20170402 10.1089/ars.2009.2974

[13] Hansson SR, Naav A, Erlandsson L. Oxidative stress in preeclampsia and the role of free fetal hemoglobin. Front Physiol. 2014;5:516 PubMed Central PMCID: PMCPMC4292435. doi: 10.3389/fphys.2014.00516 25628568 10.3389/fphys.2014.00516 PMC4292435

[14] Naav A, Erlandsson L, Axelsson J, Larsson I, Johansson M, Wester-Rosenlof L, et al A1M Ameliorates Preeclampsia-Like Symptoms in Placenta and Kidney Induced by Cell-Free Fetal Hemoglobin in Rabbit. PLoS One. 2015;10(5):e0125499 PubMed Central PMCID: PMCPMC4425457. doi: 10.1371/journal.pone.0125499 25955715 10.1371/journal.pone.0125499 PMC4425457

[15] Ozolek JA. Cord Blood Hemoglobin Screening: Normal Values, Sex Differences, Is it Necessary? 1314. Pediatr Res. 1998;43(S4):225.

[16] Galacteros F, Guilloud-Bataille M, Feingold J. Sex, gestational age, and weight dependancy of adult hemoglobin concentration in normal newborns. Blood. 1991;78(4):1121–4. 1868243

[17] Banerjee J, Asamoah FK, Singhvi D, Kwan AW, Morris JK, Aladangady N. Haemoglobin level at birth is associated with short term outcomes and mortality in preterm infants. BMC Med. 2015;13:16 PubMed Central PMCID: PMCPMC4307132. doi: 10.1186/s12916-014-0247-6 25622597 10.1186/s12916-014-0247-6 PMC4307132

[18] Brown RN. Maternal adaptation to pregnancy is at least in part influenced by fetal gender. BJOG. 2015. 10.1111/1471-0528.13529 26224389

[19] Walker MG, Fitzgerald B, Keating S, Ray JG, Windrim R, Kingdom JC. Sex-specific basis of severe placental dysfunction leading to extreme preterm delivery. Placenta. 2012;33(7):568–71. doi: 10.1016/j.placenta.2012.03.011 22513321 10.1016/j.placenta.2012.03.011

[20] Chu T, Bunce K, Shaw P, Shridhar V, Althouse A, Hubel C, et al Comprehensive analysis of preeclampsia-associated DNA methylation in the placenta. PLoS One. 2014;9(9):e107318 PubMed Central PMCID: PMCPMC4172433. doi: 10.1371/journal.pone.0107318 25247495 10.1371/journal.pone.0107318 PMC4172433

[21] Elsmen E, Kallen K, Marsal K, Hellstrom-Westas L. Fetal gender and gestational-age-related incidence of pre-eclampsia. Acta Obstet Gynecol Scand. 2006;85(11):1285–91. doi: 10.1080/00016340600578274 17091404 10.1080/00016340600578274

[22] Global Pregnancy C, Schalekamp-Timmermans S, Arends LR, Alsaker E, Chappell L, Hansson S, et al Fetal sex-specific differences in gestational age at delivery in pre-eclampsia: a meta-analysis. Int J Epidemiol. 2016. 10.1093/ije/dyw178 PMC5837300 27605586

[23] Garnica AD, Chan WY. The role of the placenta in fetal nutrition and growth. J Am Coll Nutr. 1996;15(3):206–22. 8935436 10.1080/07315724.1996.10718591

[24] Barcena A, Muench MO, Kapidzic M, Fisher SJ. A new role for the human placenta as a hematopoietic site throughout gestation. Reprod Sci. 2009;16(2):178–87. PubMed Central PMCID: PMCPMC2731631. doi: 10.1177/1933719108327621 19208786 10.1177/1933719108327621 PMC2731631

[25] Robin C, Bollerot K, Mendes S, Haak E, Crisan M, Cerisoli F, et al Human placenta is a potent hematopoietic niche containing hematopoietic stem and progenitor cells throughout development. Cell Stem Cell. 2009;5(4):385–95. PubMed Central PMCID: PMCPMC2812802. doi: 10.1016/j.stem.2009.08.020 19796619 10.1016/j.stem.2009.08.020 PMC2812802

[26] Dzierzak E, Robin C. Placenta as a source of hematopoietic stem cells. Trends Mol Med. 2010;16(8):361–7. PubMed Central PMCID: PMCPMC3586314. doi: 10.1016/j.molmed.2010.05.005 20580607 10.1016/j.molmed.2010.05.005 PMC3586314

[27] Ottersbach K, Dzierzak E. The placenta as a haematopoietic organ. Int J Dev Biol. 2010;54(6–7):1099–106. doi: 10.1387/ijdb.093057ko 20711987 10.1387/ijdb.093057ko

[28] Van Handel B, Prashad SL, Hassanzadeh-Kiabi N, Huang A, Magnusson M, Atanassova B, et al The first trimester human placenta is a site for terminal maturation of primitive erythroid cells. Blood. 2010;116(17):3321–30. PubMed Central PMCID: PMCPMC2995359. doi: 10.1182/blood-2010-04-279489 20628147 10.1182/blood-2010-04-279489 PMC2995359

[29] Hermansen MC. Nucleated red blood cells in the fetus and newborn. Arch Dis Child Fetal Neonatal Ed. 2001;84(3):F211–5. PubMed Central PMCID: PMCPMC1721260. doi: 10.1136/fn.84.3.F211 11320052 10.1136/fn.84.3.F211 PMC1721260

[30] Wang Y, Zhao S. Vascular Biology of the Placenta San Rafael, CA: Morgan & Claypool Life Sciences; 2010. 21452443

[31] Westgate J, Rosén KG. Acid base balance at birth In: van Geijin HP, Copray FJA, editors. A Critical Appraisal of Fetal Surveillance. Amsterdam: Elsevier; 1994 p. 595–603.

[32] Tranquilli AL, Dekker G, Magee L, Roberts J, Sibai BM, Steyn W, et al The classification, diagnosis and management of the hypertensive disorders of pregnancy: A revised statement from the ISSHP. Pregnancy Hypertens. 2014;4(2):97–104. doi: 10.1016/j.preghy.2014.02.001 26104417 10.1016/j.preghy.2014.02.001

[33] Mokarami P. Pitfalls in interpreting umbilical cord blood gases and lactate at birth Sweden: Lund University; 2013.

[34] Gruslin A, Lemyre B. Pre-eclampsia: fetal assessment and neonatal outcomes. Best Pract Res Clin Obstet Gynaecol. 2011;25(4):491–507. doi: 10.1016/j.bpobgyn.2011.02.004 21474384 10.1016/j.bpobgyn.2011.02.004

[35] Akercan F, Cirpan T, Saydam G. Nucleated red blood cells in infants of women with preterm labor and pre-eclampsia. Int J Gynaecol Obstet. 2005;90(2):138–9. doi: 10.1016/j.ijgo.2005.04.019 15958265 10.1016/j.ijgo.2005.04.019

[36] Lurie S, Mamet Y. Red blood cell survival and kinetics during pregnancy. Eur J Obstet Gynecol Reprod Biol. 2000;93(2):185–92. 11074141 10.1016/s0301-2115(00)00290-6

[37] Ferber A, Fridel Z, Weissmann-Brenner A, Minior VK, Divon MY. Are elevated fetal nucleated red blood cell counts an indirect reflection of enhanced erythropoietin activity? Am J Obstet Gynecol. 2004;190(5):1473–5. doi: 10.1016/j.ajog.2004.02.033 15167873 10.1016/j.ajog.2004.02.033

[38] Teramo KA, Widness JA. Increased fetal plasma and amniotic fluid erythropoietin concentrations: markers of intrauterine hypoxia. Neonatology. 2009;95(2):105–16. PubMed Central PMCID: PMCPMC2863306. doi: 10.1159/000153094 18776724 10.1159/000153094 PMC2863306

[39] Ponder KL, Barcena A, Bos FL, Gormley M, Zhou Y, Ona K, et al Preeclampsia and Inflammatory Preterm Labor Alter the Human Placental Hematopoietic Niche. Reprod Sci. 2016;23(9):1179–92. doi: 10.1177/1933719116632926 26944948 10.1177/1933719116632926 PMC5933163

[40] Kratz A, Ferraro M, Sluss PM, Lewandrowski KB. Case records of the Massachusetts General Hospital. Weekly clinicopathological exercises. Laboratory reference values. N Engl J Med. 2004;351(15):1548–63. doi: 10.1056/NEJMcpc049016 15470219 10.1056/NEJMcpc049016

[41] Coviello AD, Kaplan B, Lakshman KM, Chen T, Singh AB, Bhasin S. Effects of graded doses of testosterone on erythropoiesis in healthy young and older men. J Clin Endocrinol Metab. 2008;93(3):914–9. PubMed Central PMCID: PMCPMC2266950. doi: 10.1210/jc.2007-1692 18160461 10.1210/jc.2007-1692 PMC2266950

[42] Bachman E, Travison TG, Basaria S, Davda MN, Guo W, Li M, et al Testosterone induces erythrocytosis via increased erythropoietin and suppressed hepcidin: evidence for a new erythropoietin/hemoglobin set point. J Gerontol A Biol Sci Med Sci. 2014;69(6):725–35. PubMed Central PMCID: PMCPMC4022090. doi: 10.1093/gerona/glt154 24158761 10.1093/gerona/glt154 PMC4022090

[43] Beggs LA, Yarrow JF, Conover CF, Meuleman JR, Beck DT, Morrow M, et al Testosterone alters iron metabolism and stimulates red blood cell production independently of dihydrotestosterone. Am J Physiol Endocrinol Metab. 2014;307(5):E456–61. PubMed Central PMCID: PMCPMC4154071. doi: 10.1152/ajpendo.00184.2014 25074984 10.1152/ajpendo.00184.2014 PMC4154071

[44] Rushton DH, Dover R, Sainsbury AW, Norris MJ, Gilkes JJ, Ramsay ID. Why should women have lower reference limits for haemoglobin and ferritin concentrations than men? BMJ. 2001;322(7298):1355–7. PubMed Central PMCID: PMCPMC1120434. 11387188 10.1136/bmj.322.7298.1355 PMC1120434

[45] Burman D. Haemoglobin levels in normal infants aged 3 to 24 months, and the effect of iron. Arch Dis Child. 1972;47(252):261–71. PubMed Central PMCID: PMCPMC1648038. 5023475 10.1136/adc.47.252.261 PMC1648038

[46] Toivanen P, Hirvonen T. Sex ratio of newborns: preponderance of males in toxemia of pregnancy. Science. 1970;170(3954):187–8. 5456613 10.1126/science.170.3954.187

[47] Hsu CD, Chan DW, Iriye B, Johnson TR, Hong SF, Repke JT. Elevated serum human chorionic gonadotropin as evidence of secretory response in severe preeclampsia. Am J Obstet Gynecol. 1994;170(4):1135–8. 8166197 10.1016/s0002-9378(94)70108-3

[48] Hsu CD, Witter FR. Fetal gender effect on preterm and term preeclamptic pregnancies. Int J Gynaecol Obstet. 1994;47(1):53–4. 7813752 10.1016/0020-7292(94)90462-6

[49] Kramer MS, Usher RH, Pollack R, Boyd M, Usher S. Etiologic determinants of abruptio placentae. Obstet Gynecol. 1997;89(2):221–6. doi: 10.1016/S0029-7844(96)00478-4 9015024 10.1016/S0029-7844(96)00478-4

